# Dementia before Death in Ageing Societies— The Promise of Prevention and the Reality

**DOI:** 10.1371/journal.pmed.0030397

**Published:** 2006-10-31

**Authors:** Carol Brayne, Lu Gao, Michael Dewey, Fiona E Matthews, Medical Research Council Cognitive Function and Ageing Study Investigators

**Affiliations:** 1 Department of Public Health and Primary Care, Institute of Public Health, Cambridge University, Cambridge, United Kingdom; 2 Medical Research Council Biostatistics Unit, Institute of Public Health, Cambridge, United Kingdom; 3 Institute of Psychiatry, King's College London, London, United Kingdom; University of Amsterdam, Netherlands

## Abstract

**Background:**

Dementia and severe cognitive impairment are very closely linked to ageing. The longer we live the more likely we are to suffer from these conditions. Given population increases in longevity it is important to understand not only risk and protective factors for dementia and severe cognitive impairment at given ages but also whether protection affects cumulative risk. This can be explored by examining the effect on cumulative risk by time of death of factors found consistently to reduce risk at particular ages, such as education and social status.

**Methods and Findings:**

In this analysis we report the prevalence of dementia and severe cognitive impairment in the year before death in a large population sample. In the Medical Research Council Cognitive Function and Ageing Study (a 10-y population-based cohort study of individuals 65 and over in England and Wales), these prevalences have been estimated by age, sex, social class, and education. Differences have been explored using logistic regression. The overall prevalence of dementia at death was 30%. There was a strong increasing trend for dementia with age from 6% for those aged 65–69 y at time of death to 58% for those aged 95 y and above at time of death. Higher prevalences were seen for severe cognitive impairment, with similar patterns. People with higher education and social class had significantly reduced dementia and severe cognitive impairment before death, but the absolute difference was small (under 10%).

**Conclusions:**

Reducing risk for dementia at a given age will lead to further extension of life, thus cumulative risk (even in populations at lower risk for given ages) remains high. Ageing of populations is likely to result in an increase in the number of people dying with dementia and severe cognitive impairment even in the presence of preventative programmes. Policy development and research for dementia must address the needs of individuals who will continue to experience these conditions before death.

## Abstract 2

The overall prevalence of dementia at death in this large study was 30%. Ageing of populations is likely to result in an increase in the number of people dying with dementia even in the presence of preventative programmes.

## Editors' Summary

**Background.:**

Severe cognitive impairment and its advanced form, dementia, are among the most difficult problems associated with aging in industrialized countries. Age-associated decline in mental functioning is also expected to become more common in developing countries as improvement of conditions that affect health leads to longer life expectancies. Although the risk of cognitive impairment is known to increase with age, the number of people who suffer from loss of mental abilities in the last years of their lives has not been well studied, as such persons are usually reported to have died from other causes. Further, because the very elderly are seldom included in prevention studies, it is not known whether factors found to reduce the risk of developing dementia by a given age will provide protection until the end of life.

**Why Was This Study Done?:**

This study was designed to follow a representative population of aged people over several years to estimate the risk of developing cognitive impairment or dementia near the end of life and to determine whether factors such as education and social class, which may be protective earlier in life, can ultimately prevent decline in mental functioning.

**What Did the Researchers Do and Find?:**

Using standardized assessments of cognitive status, the researchers interviewed people age 65 and over at six sites representing rural and urban areas in the United Kingdom. Interviews were conducted at regular intervals over ten years. Of approximately 12,000 study participants who had died by the time of this report, just over 2,500 had an assessment for dementia within one year before dying. Of this group, those who died between ages 65 and 69 had a 6% chance of dying with dementia, and those who died above age 95 had a 58% chance of dying with dementia. When moderate and severe cognitive impairment were considered together, the rate in people above age 95 reached almost 80%. Women were more likely to develop dementia than men, even after taking into account the fact that women tend to live longer than men. A higher level of education was associated with only a slightly lower risk of dementia before death.

**What Do These Findings Mean?:**

According to these results, as the number of aged persons increases (with improved health care, preventive medicine, and healthier lifestyles), the chances of developing dementia in the last years of life will continue to increase. Factors believed to protect against dementia at earlier times may be of little effect at the end of life. Planning for aging societies must therefore include not only research into treatments and preventive efforts to reduce the impact of dementia at the end of life, but also realistic allocation of resources to support individuals and their caregivers who must deal with the difficulties of cognitive decline.

**Additional Information.:**

Please access these Web sites via the online version of this summary at http://dx.doi.org/10.1371/journal.pmed.0030397.

- Web site of the Medical Research Council Cognitive Function and Ageing Study
- Web site of the Alzheimer's Association
- Wikipedia entry on dementia (note: Wikipedia is a free Internet encyclopedia that anyone can edit)

## Introduction

Changing global population structures require societies to face major issues raised by increasing numbers of the very old. Western societies have experienced sufficient change to be able to anticipate patterns of health and ill health in their ageing populations. These changes are likely to be echoed in a shorter time frame over the next decades in the less wealthy regions of the world because of their more rapid experience of demographic shift. This continuing change in life expectancy has been attributed to improved health and lower morbidity in early life, and to effective primary, secondary, and tertiary prevention in later life. Reductions in incidence have been seen in vascular disease, including cerebrovascular disease [1,2].

Increased life expectancy and improvement in many areas of health have been demonstrated, but sharp increases of morbidity with age are still observed in all populations [3]. Dementia and severe cognitive impairment are amongst the disorders with greatest increase with age in both incidence and prevalence [4,5]. Preventive action at the population level ideally eradicates risk of disease, but in reality much prevention reduces disease at a given age rather than eradicating its occurrence. The overall contribution of primary and secondary prevention to the reduction in cardiovascular disease mortality has been estimated at around half of the effect, with improved care for established disease resulting in the remaining improvement [6]. There is a global effort aimed at improving health in our older populations, and much of this effort hinges on the hope that extension of life expectancy will not be accompanied by increases in morbidity but by compression of morbidity [7].

Many of the factors that have been observed to be associated with reduced risk for chronic disease such as cancer and heart disease are also associated with lower mortality [8–10]. More recently such factors have also been studied in relation to Alzheimer disease and cerebrovascular disease and have been reported as associated with reduction in risk within the periods of follow-up [11]. These variables are often associated with social class and educational level, whose effects may be attributed to healthier lifestyles and health practices. Social class and education can be seen as proxies for these factors, and higher social class and more education have been shown in many studies to be associated with reduction in dementia outcomes [12,13].

At the same time as this attention to healthy ageing there is increasing attention to the end of life. Many people express a fear of dementia before death as well as a desire to remain independent up to the time of death rather than have prolonged periods of dependency. Quality of life in the period leading up to death is a relatively neglected area in the major emphases of most countries' health services, which tend, understandably, to focus on prevention and treatment. There is already clear evidence that individuals who have dementia and cognitive impairment have an excess mortality [14] and a shorter life expectancy [15] than individuals without. However, there is little information about the proportion of the population at any given age who die with dementia or severe cognitive impairment. Individuals at the end of life are likely to be underrepresented in studies of risk with infrequent follow-up as attrition is well known to be associated with cognitive decline and age [16]. Information on the dementia status of individuals from death certificates is inaccurate and cannot be used as a substitute [17]. More knowledge about the status of individuals in the period before and at death is important to assess the full implications of global population ageing and the potential for prevention of dementia and cognitive decline in the future. If we change risk in the population, can we expect to see different profiles in our very old population, including the period close to death? What is the association of dementia and cognitive decline with death (terminal decline), and how much can we estimate that we would avoid if all the preventive actions in place were able to effect a reduction in health inequalities? Longitudinal studies with sufficient follow-up and data on the mortality of all individuals are required to investigate dementia at death. Repeated interviews are necessary to correctly classify dementia at death. Notification of death for the whole cohort irrespective of continuing participation is also required.

The analysis presented in this paper investigates the prevalence of dementia and severe cognitive impairment in the period before death in the population-based Medical Research Council Cognitive Function and Ageing Study (MRC CFAS). It also investigates whether those who, on the basis of the literature to date, would be expected to have a relative advantage in risk for dementia at any given age are markedly less likely to die with dementia or severe cognitive impairment than those at greater risk.

## Methods

### Study Design and Population

MRC CFAS is a longitudinal population-based cohort study that involves six different study centres. The six centres were chosen because they represent the main national variation with regards to urban–rural differences, the north–south and east–west gradients, and variation in socio-economic levels and in known rates of chronic disease. All centres had existing researchers who were interested in population-based studies of the elderly. Urban sites included Liverpool, Newcastle, Nottingham, and Oxford. Rural sites included Cambridgeshire and Gwynedd, in North Wales. All centres have been used for this analysis.

The Liverpool study is described in full elsewhere [18]; a population stratified by 5-y bands over the age of 65 y was interviewed from 1989 onwards. Three full waves of follow-up (each 2 y apart) have been undertaken and used in the following analysis.

The full study design of the other five centres is described in detail elsewhere and is explained briefly here [4]. For these five centres, a total of 13,004 individuals aged 65 y and over were recruited, and interviewed at baseline (1990–1992) with a schedule including socio-demographic items, general health items, and cognitive items. Participants have been re-interviewed at various times, either the whole cohort or sub-samples stratified by centre, age, and cognitive state. Three types of interviews were used during the study. Baseline examination was conducted via a screening interview. A median of 3 mo after a screening interview, 20% of individuals had a diagnostic interview (assessment interview). The assessment interview consisted of a participant interview and an informant interview. The former consisted of the Geriatric Mental State (GMS), version B3, from which AGECAT [19] could be derived. The latter consisted of the History and Aetiology Schedule [20]. These are standardised psychiatrically based interviews to establish an algorithmic clinic diagnosis described below. Incidence screen and assessments and combined screen and assessment interviews were used during the follow-ups at 2 y, and combined screen and assessment interviews on all participants at 10 y. All interviews contained the Mini-Mental State Examination (MMSE). The Cambridge centre additionally interviewed the complete sample with a combined screen and assessment interview at 6 y. In Liverpool the whole population was interviewed with the full GMS AGECAT algorithm and the clinical version of the MMSE [21] on all these occasions. A study diagnosis of dementia and cognitive impairment can be derived from successful interviews.

All participants were flagged for mortality information. Death information was obtained from the Office of National Statistics up to the end of 2004. Version 8.0 of the five-centre identical data and version c4 for the Liverpool data have been used.

### Definition of Dementia

The study diagnosis of dementia is based on the GMS AGECAT algorithm. This algorithm has been validated against clinicians and DSM-III-R [22]. In addition, for the analysis presented here, organicity level and memory defect rating at the last available interview before death were combined with ICD-10 diagnosis [23] (F00–F03, F29, F051, or G30) on the death certificate. Misclassification with organicity due to transient delirium is rare in the population studies [4].

### Definition of Cognitive Impairment

MMSE scores were coded within each centre using identical methodology (ignoring backwards WORLD and coding “not answered” responses to incorrect). Some differences existed between the implementation of the questions (for example, apple, table, and penny in the five identical centres was any three items in Liverpool); however, examination of the distribution of the scores indicates that they have similar distributional properties at the lower end. The scores were grouped into severe cognitive impairment (MMSE < 18) or not, and moderate/severe cognitive impairment (MMSE < 22). Where the MMSE was missing at the last interview before death, a previous interview was used if cognitive impairment had already been demonstrated. In the analysis presenting cognitive impairment, the classification of dementia status on death certificates was ignored. Individuals with cognitive impairment were classified only from interview responses, and hence some individuals with dementia on death certificates were not classified as cognitively impaired.

### Interview Waves

Only study waves where the complete population was interviewed were used in this analysis. Hence, information for Liverpool years 0, 2, and 4 and for the other five centres years 0, 2, 6 (Cambridge only), and 10 were used.

The dementia or cognitive impairment status required is the status at death for an individual, hence only those individuals whose last interview was within 1 y of their death were considered. A sensitivity analysis expanding this to deaths within 2 y has been undertaken, which is based on more deaths, but which underestimates the true amount of dementia/cognitive impairment.

### Statistical Methods

Prevalence of dementia at death was calculated assuming binomial proportions with exact confidence intervals (CIs). Logistic regression analysis was used to investigate the relationships between confounders and prevalence of dementia at death. All analysis was undertaken using STATA version 8.0 (Stata Corporation, College Station, Texas, United States).

## Results

### Deaths

A total of 4,319 individuals from Liverpool (from an original sample 5,244) and 8,068 individuals from the other centres (from 13,004)—altogether 12,387 individuals—died by the time of this analysis. Of these, 12,286 deaths (99%) have information available from their death certificate. A total of 2,566 individuals who had a known dementia status at last interview died within 1 y of this interview, and 5,053 died within 2 y of this interview. A total of 2,555 individuals are included in the analysis of severe cognitive impairment, and 2,573 individuals in the analysis of moderate/severe cognitive impairment.

### Dementia at Death

**Figure 1:**
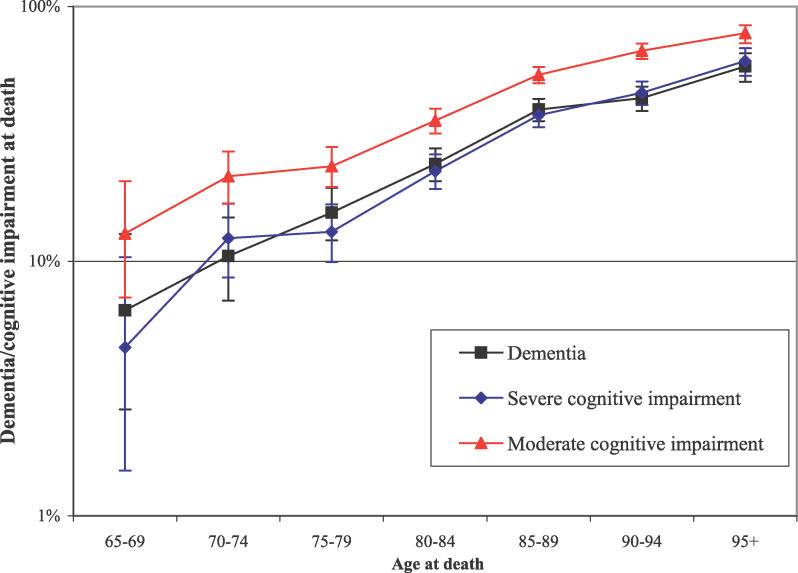
Dementia or Cognitive Impairment by Age at Death with 95% CIs

Table 1 shows a summary of dementia status at death classified by centre, sex, social class, education level, and age at death. Figure 1 shows a sustained increase in the proportion of individuals receiving a study diagnosis of dementia in the period before death with increased age at death (log). People who died between the ages of 65 and 69 y had a 6% risk of dying with dementia, but people who died above age 95 y had an over 58% risk of dying with dementia. There are wide confidence intervals around the estimate for the 31 individuals aged over 100 y at death, compatible with continued increase and stabilisation (42% demented, 95% CI 25%–61%).

**Table 1:**
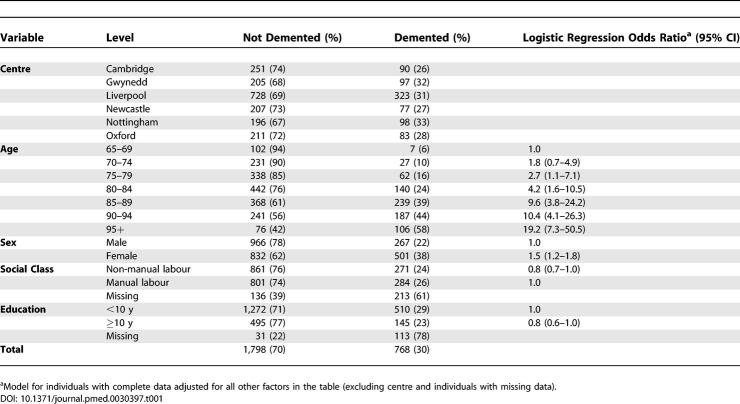
Prevalence of Dementia at Death and Relationship to Potential Risk Factors for All Individuals Who Died within 1 y of Last Interview

**Figure 2:**
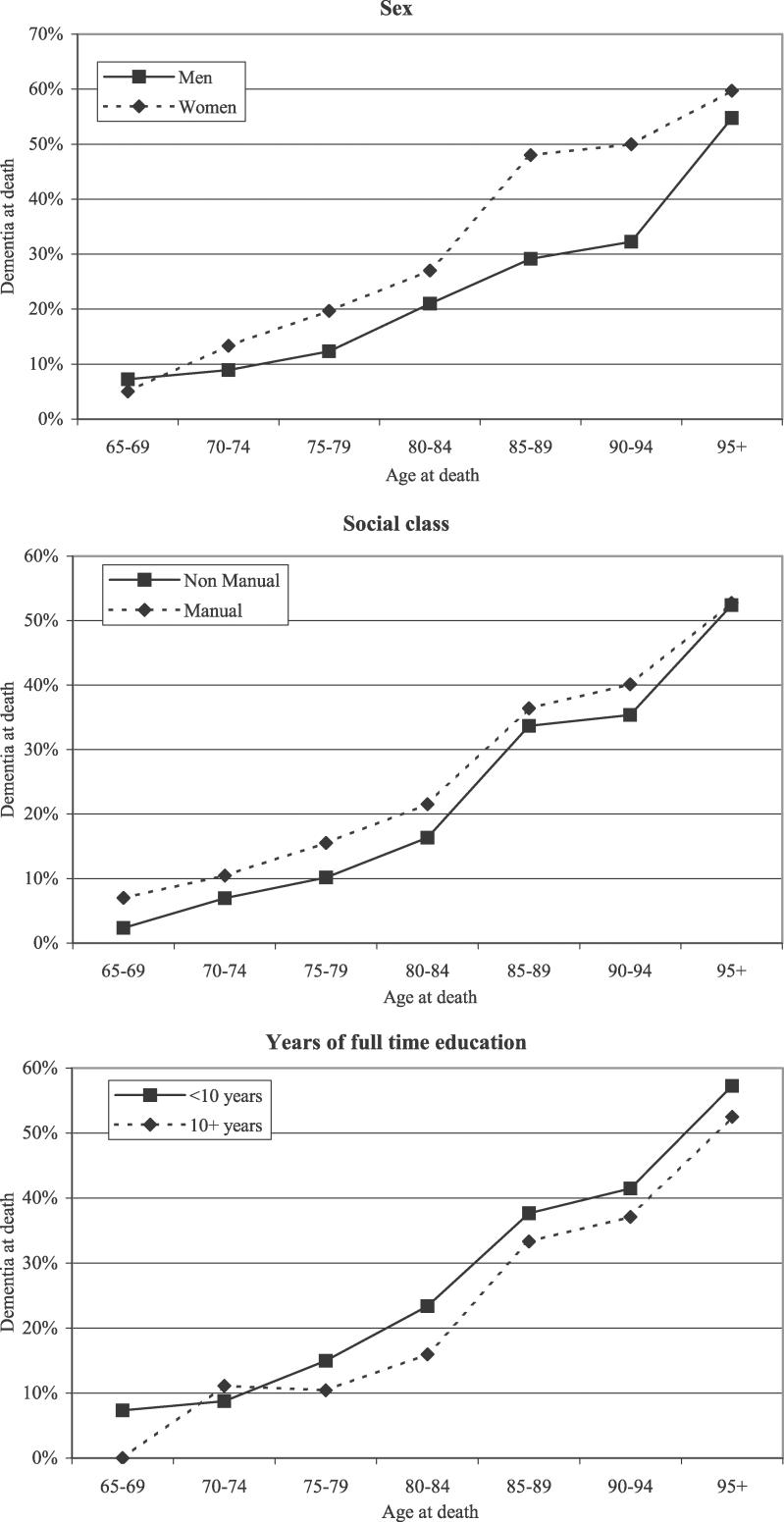
Rate of Dementia at Death by Sex, Education, and Social Class

There was a gender difference in risk, with women demonstrating an overall prevalence of dementia before death of 38%, almost double that of men. However, women have a longer life expectancy than men [24], and dementia prevalence increase with age [4]. Adjusting for age showed that women still had a higher absolute proportion of individuals with dementia than men (Table 1; Figure 2); however, there was no evidence that the effect of age differed in men and women (likelihood ratio test for interaction, χ^2^ = 7.6, *p* = 0.27). Dementia before death was less common in the non-manual-labour social classes than in the manual-labour group at all ages, but not after adjusting for other factors (*p* = 0.09) (Table 1; Figure 2). The absolute difference at death was 2%. A higher level of education was associated with slightly lower prevalence of dementia before death, even after adjusting for the other factors (*p* = 0.02) (Table 1; Figure 2). The individuals who had 10 y or more of full-time education had slightly lower dementia prevalence than individuals with less than 10 y of full-time education. The absolute difference between these educational levels was 6%. There was no evidence of any interaction between sex, education, and social class (likelihood ratio tests all had *p* > 0.2). An individual analysis investigated whether the age effect was partially explained by different patterns of dementia seen in different birth cohorts. There was no evidence that suggested there has been a change in the age and sex pattern by birth cohort after adjusting for all other covariates (likelihood ratio test, *p* > 0.2).

Those participants who had an unknown social class and/or educational level (378, 15%) were more likely to have been demented at both baseline and follow-up (62% demented). A sensitivity analysis including these missing values as either the highest or lowest categories did not alter the findings.

### Cognitive Impairment

The proportion of individuals with severe cognitive impairment before death was very similar to the proportion of individuals with dementia at death, with the same marked increase in cognitive impairment before death with age (Figure 1; Table 2). When individuals with moderate or severe cognitive impairment were included together, the rate was around 10% higher at all ages, with nearly 80% of individuals dying over 95 y of age having this level of cognitive impairment.

**Table 2:**
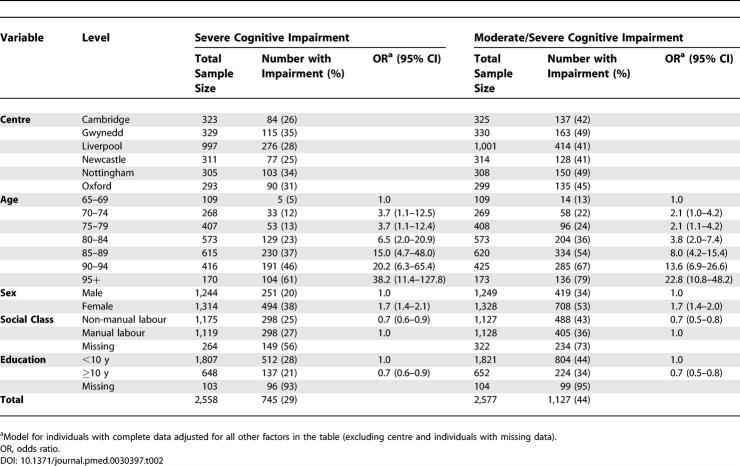
Prevalence of Moderate and Severe Cognitive Impairment at Death and the Relationship with Potential Risk Factors

The relationship between cognitive impairment and demographic risk factors was almost identical between the less and more inclusive levels of impairment and similar to the relationships with dementia at death, though the effects were all slightly stronger. The patterns were also similar if individuals with dementia were removed from the analysis (unpublished data). The absolute difference in severe cognitive impairment at death between social class groups was 5% and for educational levels was 7%. The absolute differences for moderate/severe cognitive impairment were 7% and 10%, respectively.

### Centenarians

There were 31 centenarians in the study who died within 1 y of interview. Of these, 25 (81%) were female, 13 (42%) had dementia, and 13 (46%) had severe cognitive impairment (with three missing information).

### Centre Effects

Although not the primary purpose of this analysis, centre was included as it may indicate unmeasured confounders. For dementia there were no differences between the centres once age and sex were accounted for. There were some small differences between centres for cognition before death, but no consistent pattern emerged.

### Sensitivity Analysis

Including all individuals with an interview within 2 y of death did lower the proportions of individuals with dementia before death slightly (27% versus 30%). The proportion with dementia within 1 y of death (30%) was higher than that estimated for individuals seen between 1 and 2 y before death (23%; *t*-test for difference, *p* < 0.001), showing the proximity of much dementia to death. The findings in relation to age, sex, social class, and education were similar. Excluding individuals with limited information (such as screen-only data) did not indicate introduction of bias.

### Population Impact

These results were combined with population projections for mortality. The number of deaths per year by age for England and Wales and the US [25,26] was combined with the prevalence of dementia at death. The prevalence rate of dementia was assumed to stay constant with time as there has been no apparent change with each successive 5-y birth cohort studied (year of birth ranges from 1888 to 1928). The results indicate that currently there are 114,000 individuals (95% CI 97,000–134,000) in England and Wales and 487,000 individuals (95% CI 362,000–503,000) in US who die with dementia each year. In 20 years' time, these numbers will have increased to 138,000 (95% CI 118,000–160,000) in England and Wales and 528,000 (95% CI 443,000–627,000) in the US.

## Discussion

### Summary of Findings

This 10-y longitudinal population study of people aged 65 y and over at baseline examines dementia and severe cognitive impairment status in the period before death and shows that the prevalence of dementia and severe cognitive impairment in the period before death rises steeply with age. Thus, by the time an individual aged 90 y dies, the risk of being demented or severely cognitively impaired is around 60%. Investigating whether higher education and social class—proxies for healthy exposures and lifestyles—protected individuals from dementia by the time they died showed that individuals with higher education and social class were at significantly reduced risk of dying with dementia or cognitive impairment. However, this reduction was limited: the reduction in dementia had an absolute value of only 2% and 7%, respectively, for higher social class and more highly educated groups; the reduction in cognitive impairment was 7% and 10%, respectively; and both high education and social class together conferred a reduction of 7% for dementia and 10% for cognitive impairment. Thus, the inequalities in healthy lifestyles and social advantage observed at given ages appear to be attenuated by the longer life expectancy of those individuals.

### Critique of Findings

There are features of the study that need to be taken into account in interpreting the findings. The response rate at baseline and at each of the follow-up interviews was around 80% (see http://www-cfas.medschl.cam.ac.uk for details of audit trail). Some bias could be introduced into the analysis through this drop out due to death, refusal, and moving away. We know from earlier analysis of the dataset that individuals with cognitive impairment are at greater risk of death, so there is some potential to underestimate the prevalence of dementia at death given that we do not have cognitive status close to death in all respondents. We have taken this into account through the analysis of only those individuals who died within a relatively short period of being interviewed. If the analysis is conducted on the total sample for whom the last interview was more distant from death, a lower prevalence of dementia and cognitive impairment is found, as expected. However, individuals who dropped out from the study in the year preceding death were no more likely to be demented than those who undertook an interview in this time, indicating that the individuals analysed here are unlikely to be biased.

The measure for dementia in this study was GMS diagnostic algorithm organicity status, and for cognitive impairment was MMSE. Both of these will suffer from some misclassification bias. We have found severe MMSE and dementia states to be relatively stable, with only small proportions moving out of these categories in the extensive longitudinal data available. Limiting the analysis to subtypes of dementia such as Alzheimer disease or vascular dementia might indicate preventive potential for specific subtypes by death but would not reveal the true population picture, nor does cognitive impairment and dementia in the oldest old tend to fit into pure diagnostic categories [27]. Moreover, the concern many older people express is not about specific forms of dementia but that they will lose their independence and become cognitively frail. We have not addressed the mildest stages of impairment in this analysis.

Examining education and social class as proxies for healthy lifestyles could be seen as a crude approach, but these have been shown in their own right, in some studies, to be independently associated with lower rates of dementia. Certainly it would be desirable to expand this type of analysis to population studies with better lifestyle and health indicators—peak physical fitness, no smoking, moderate alcohol, good nutrition, and no hypertension, diabetes, or heart disease.

There are very few studies with which to compare these findings. One US study examined patterns of change in functional disability in the period before death [28]. Its goal was more to examine the predictive value of such patterns. It is already well established that cognitive impairment and dementia are associated with increased risk of death. We set out to examine how common dementia is by the time of death and whether those groups who are held to be at reduced risk of dementia and chronic disease during life appear to escape the risk before death. We have found that this is not the case, although there is a small diminution in risk before death, consistent with the absolute risk reductions reported for given ages in cohort studies. These findings confirm the finding of an earlier study by Bickel, who conducted a retrospective study of deaths by asking relatives about the status of the deceased before death and reported a prevalence of dementia-related conditions at death even higher than our estimates [29]. Our data also agree with findings on the health state of the very old, including centenarians. MRC CFAS reports odds ratios of incidence of dementia of over 25 for the 85-y-and-over age group compared to the 65- to 69-y age group [30]. In a study of 34 centenarians by Silver and colleagues [31], most (64%) were demented. Systematic reviews have shown that prevalence of dementia at this age is between 50% and 64% [32], with only 15%–25% estimated to have functionally intact cognition [33,34].

### Implications and Conclusions

These data have serious implications for societies that are ageing. All public health services internationally will and should continue to promote healthy ageing through healthy lifestyles and optimal health care. It is important to continue to research treatments and preventive approaches for specific dementias aimed at lessening the devastation that dementia and cognitive impairment can cause. Substantial claims have been made for the prevention of dementia, and these need to be backed up by robust evidence of risk reduction through preventive strategies—not yet available as the evidence is based on observational studies. However, our findings suggest that preventive efforts are unlikely to be able to counteract the profound effects of age and proximity to death altogether. We will continue to examine early risk and track cognitive change, but the analysis presented here is about quality of life at the end of life. It may be that, although there will be a preventable component to dementia giving us a small and important absolute reduction in expectation of dementia at given ages, there is also a component that is not amenable to such types of prevention [35]. Researchers may be doing those who are ageing now and themselves a disservice in the future if they assume, and project to the public, that dementia and cognitive impairment can be prevented altogether during increasingly long lives. Given that the population of all deaths accounted for by people aged 85 y and over has increased from 8% in 1960 to 21% in 2003 in men and 16% to 41% in women in England and Wales, this is a matter of considerable importance [36]. Our data show that in the UK (and potentially in the US) the burden of dementia and cognitive impairment at time of death is substantial and is likely to become an increasing problem as the population ages. It is essential and urgent that societies invest in planning for (and researching) quality at the end of life for those with dementia and severe cognitive impairment [37–39], with sensible estimation of the likely increase in the numbers affected in the decades to come.

## Abbreviations

CI: confidence interval
GMS: Geriatric Mental State
MMSE: Mini-Mental State Examination
MRC CFAS: Medical Research Council Cognitive Function and Ageing Study
